# SS-31 Protects Liver from Ischemia-Reperfusion Injury via Modulating Macrophage Polarization

**DOI:** 10.1155/2021/6662156

**Published:** 2021-04-13

**Authors:** Longcheng Shang, Haozhen Ren, Shuai Wang, Hanyi Liu, Anyin Hu, Peng Gou, Yunzhen Lin, Jingchao Zhou, Wei Zhu, Xiaolei Shi

**Affiliations:** ^1^Department of Hepatobiliary Surgery, The Affiliated Drum Tower Hospital of Nanjing University Medical School, Nanjing, China; ^2^Hepatobiliary Institute Nanjing University, Nanjing, China; ^3^Department of Anesthesiology, The Affiliated Drum Tower Hospital of Nanjing University Medical School, Nanjing, China

## Abstract

Ischemia-reperfusion injury (IRI) is a common complication in liver surgeries. It is a focus to discover effective treatments to reduce ischemia-reperfusion injury. Previous studies show that oxidative stress and inflammation response contribute to the liver damage during IRI. SS-31 is an innovated mitochondrial-targeted antioxidant peptide shown to scavenge reactive oxygen species and decrease oxidative stress, but the protective effects of SS-31 against hepatic IRI are not well understood. The aim of our study is to investigate whether SS-31 could protect the liver from damages induced by IRI and understand the protective mechanism. The results showed that SS-31 treatment can significantly attenuate liver injury during IRI, proved by HE staining, serum ALT/AST, and TUNEL staining which can assess the degree of liver damage. Meanwhile, we find that oxidative stress and inflammation were significantly suppressed after SS-31 administration. Furthermore, the mechanism revealed that SS-31 can directly decrease ROS production and regulate STAT1/STAT3 signaling in macrophages, thus inhibiting macrophage M1 polarization. The proinflammation cytokines are then significantly reduced, which suppress inflammation response in the liver. Taken together, our study discovered that SS-31 can regulate macrophage polarization through ROS scavenging and STAT1/STAT3 signaling to ameliorate liver injury; the protective effects against hepatic IRI suggest that SS-31 may be an appropriate treatment for liver IRI in the clinic.

## 1. Introduction

Ischemia-reperfusion injury (IRI) during liver resection and transplantation is an acute process that occurs after artery reopening. It is mainly characterized by local sterile-inflammation response [1]. If not processed in an appropriate manner, IRI could lead to hepatocellular death and increase the risk of acute or chronic transplant rejection [2]. The mechanism underlying hepatic IRI is still not fully understood, and there is no effective management available for IRI.

Oxidative stress, which is caused by excess generation of ROS, plays a key role during IRI. When the blood supply recovers, a large amount of ROS is generated from mitochondria during the early phase of IRI [3], which then leads to mitochondrial dysfunction [4]. Accumulation of ROS is highly cytotoxic, causing cell death, disruption of microvasculature integrity, and inflammation response [5]. Abundant evidences have suggested that antioxidants might be an alternative strategy for treating IRI *in vitro* and *in vivo* [6–8]. For instance, caffeic acid, an effective antioxidant derived from many plants in nature, has been shown to reduce the oxidative stress and microcirculatory disturbance caused by hepatic IRI through regulating the mitochondrial respiratory chain [8].

The inflammation response induced by cascades of free radicals plays a pivotal role in hepatic IRI [9, 10]. Indeed, the degree of tissue damage mostly depends on the duration of ischemia and the magnitude of inflammation response compromised of macrophage and neutrophil infiltration [11]. Macrophage is a major component of the innate immune system. The activation of macrophages has two modes, classical activation (M1) and alternative activation (M2) [12]. The M1-type macrophages marked by TNF*α*, IL1*β*, and iNOS contribute to proinflammation and tissue destruction, while the M2-type macrophages marked by CD206, IL10, and Arg1 promote anti-inflammation and tissue regeneration [13, 14]. In some liver diseases, such as alcoholic liver disease (ALD) [15] and nonalcoholic fatty liver disease (NAFLD) [16], modulating macrophage polarization and function has been recognized as a potential therapeutic strategy. A recent study reported that Roquin-1 could inhibit macrophage M1 polarization via suppressing the AMPK/mTOR/STAT3 pathway, thus reducing the liver IRI [17]. Our previous study also demonstrated that regulating macrophage polarization could alleviate the liver damage caused by IRI in mice with NAFLD [18]. Therefore, understanding the mechanism of innate immune activation, especially the modulation of macrophage phenotype transformation, is important for developing novel therapeutic strategies to alleviate liver inflammation and damage in hepatic IRI.

SS-31 is a new and promising antioxidant peptide targeting mitochondria, which was developed by Zhao et al. in 2004 [19]. SS-31 can improve ATP production, reduce mitochondrial ROS production [20], and interact with mitochondrial cardiolipin [21], thus decreasing oxidative stress under multiple conditions. Due to its antioxidant activity, SS-31 has been applied in treating Alzheimer's disease [22], Parkinson's disease [23], atherosclerotic [24], and IRI [25–27]. In our previous study, we also demonstrated that SS-31 could protect macrophages from foam cell formation through decreasing ROS and inhibiting cholesterol influx [28]. In addition, SS-31 has various advantages that can support its future clinical applications. For example, SS-31 is a small polypeptide that is water-soluble and cell-permeable; moreover, SS-31 targets the inner membrane of mitochondria and prevents mitochondrial depolarization [29].

So far, the role of SS-31 in hepatic IRI is still unclear. We hypothesized that SS-31 could ameliorate hepatic IRI through reducing inflammation response and oxidative stress, similar to the way that it alleviates injury in other organs. In this study, we reported that SS-31 could modulate macrophage polarization to protect the liver from IRI. These results may provide a new approach for treating hepatic IRI during liver surgical procedures.

## 2. Materials and Methods

### 2.1. Animals

The C57BL/6J mice used in this study were purchased from the Animal Center of Nanjing Drum Tower Hospital. The mice were 6-8 weeks old and about 20 g in weight. 36 male mice were divided into 4 groups, which were the sham, sham+SS-31, IRI+vehicle, and IRI+SS-31 groups (*n* = 5 per group for collecting samples and *n* = 4 per group for isolating KCs). The mice were housed under specific pathogen-free conditions. All the animal experiments were approved by the Institutional Animal Care and Use Committee of Nanjing University, China, based on the NIH Guidelines for the Care and Use of Laboratory Animals. Measures were taken to minimize the distress and pain experienced by mice.

### 2.2. Reagents

SS-31 peptide (purity 97.42%) was synthesized by ChinaPeptides (Shanghai, China). LPS (L2630) was purchased from Sigma-Aldrich (USA). The primary antibodies used in this study included TNF*α* (Santa Cruz, sc12744), iNOS (Abcam, ab178945), IL1*β* (Abcam, ab234437), 8-OHDG (Santa Cruz, sc393871), *β*-actin (ProteinTech, 60008-1-Ig), Nrf2 (Cell Signaling Technology,12721t), HO-1 (Abcam, ab189491), SOD2 (Abcam, ab110300), STAT1 (Cell Signaling Technology, 14994s), p-STAT1 (phospho Tyr701) (Cell Signaling Technology, 9167s), STAT3 (Cell Signaling Technology, 12640s), and p-STAT3 (phospho Tyr705) (Cell Signaling Technology, 9145s). The antibodies were used according to the manufacturer's instructions.

### 2.3. Cell Culture and Treatment

Raw264.7 cells were purchased from ATCC and cultured in RPMI 1640 medium supplemented with 10% FBS, penicillin (100 U/mL), and streptomycin (100 mg/mL). The cells were cultured in a 37°C incubator with 5% CO_2_. When cell density reached 60%-70%, they were treated with LPS (100 *μ*g/mL) with or without SS-31 (20 nM) for 24 h.

KCs were isolated as previously described [30]. Briefly, the mice were anesthetized with isoflurane and their abdomens were cut open. The liver was perfused with CMF-HBSS containing 0.02% type IV collagenase via the portal vein until the liver turned pale. Then, the liver was removed gently and digested in 0.02% type IV collagenase for 30 minutes. To remove undigested tissue and connective tissue, the liver was dissociated and the cell suspension was filtered through a sterile 70 *μ*m nylon strainer and centrifuged at 300g for 5 min twice to collect nonparenchymal cells (NPCs). Then, NPCs were resuspended in HBSS, and a 50/25% Percoll gradient was used to separate KCs. The KCs in the middle layer were collected and cultured in RPMI 1640 with 10% FBS and 1% antibiotics for 1 day, and the unattached cells were removed.

### 2.4. Liver IRI Model

We used an established mouse model of liver warm ischemia-reperfusion injury as previously described [31]. Briefly, the mice were anesthetized by isoflurane, fixed on the platforms, and disinfected with povidone-iodine. The arterial blood vessels and portal veins were clamped to block blood flow in 70% of the mouse liver (left and middle lobes). After 60 min of ischemia, clamps were removed. The mice were sacrificed with high-dose isoflurane after 6 h of reperfusion. Blood samples were collected upon scarification for further analysis. The liver tissues were cut into pieces and fixed in 4% formalin or snap frozen in liquid nitrogen; SS-31 (5 mg/kg, in saline) or vehicle (saline, 200 mL) was intraperitoneally injected at 1 h prior to surgery. The dose and injection time were chosen based on our previous studies [18, 32] or other published literature [25, 33].

### 2.5. Western Blotting

Western blot assays were conducted as previously described [28]. Proteins were extracted from cells or tissues using RIPA Lysis Buffer (KeyGEN Biotech, China). The concentration of proteins was determined by BCA assay (KeyGEN Biotech, China) following the manufactures' instruction. Proteins (20 *μ*g) were denatured at 100°C for 10 min in sample buffer containing SDS and *β*-mercaptoethanol. The samples were subjected to electrophoresis by SDS/PAGE (12% and 10% gel), and the blots were incubated primary antibodies mentioned above at 4°C overnight. The next day, the blots were incubated with corresponding HRP-conjugated secondary antibodies (ProteinTech, China) at room temperature for one hour and then subjected to substrate development.

### 2.6. Quantitative Real-Time PCR

Total RNA was extracted from KCs and macrophages with TRIzol™ (Life Technologies, USA) following the manufacturer's instructions.

Reverse transcription was performed with HiScript II RT SuperMix for qPCR+gDNA wiper (R223-01, Vazyme Biotech, China), and qPCR was performed in triplicates with ChamQ SYBR qPCR Master Mix (Q331-02, Vazyme Biotech, China) and the ABI PRISM 7500 Real-Time PCR System (Applied Biosystems, USA). The sequences of qPCR primers are listed in Supplementary Table 1. ACTB was used as the internal reference gene.

### 2.7. ELISA

The concentrations of cytokines TNF*α* (MultiSciences, China), IL6 (MultiSciences, China), IL10 (eBioscience, USA), and IL1*β* (MultiSciences, China) in serum were measured by commercial ELISA kits according to the manufacturers' instructions.

### 2.8. Histological and Immunohistochemical Analysis

Paraffin-embedded sections were stained with hematoxylin and eosin (HE) to evaluate liver damage caused by IRI, and images were acquired by a light microscope. The degree of liver damage was evaluated according to Suzuki's score by three pathologists independently. To identify oxidative stress, the paraffin-embedded liver sections were stained with 8-OHDG as previously described [34].

### 2.9. Immunofluorescence

Immunofluorescence was performed as previously described. Briefly, frozen liver samples (4 *μ*m) were fixed by acetone for 15 min and then permeabilized with 0.3% Triton for 15 min. Afterwards, the slides were blocked with 10% fetal sheep serum for at least 1 hour, followed by incubation with primary antibodies overnight at 4°C. The slides were then washed and incubated with corresponding secondary antibodies plus DAPI for 2 hours at room temperature. The images were acquired from a fluorescent microscope. For cellular immunofluorescence staining, we seeded cells on a 24-well plate; the cells were fixed with 4% paraformaldehyde at an appropriate density, and the following steps were the same as above.

DHE (KGAF019, KeyGEN Biotech, China) staining and TUNEL (KGA67062, KeyGEN Biotech, China) staining were performed to evaluate oxidative stress and apoptosis in the liver, respectively, and the procedures followed the manufacturers' instructions.

DCFH-DA (S0033S, Beyotime, China) were used to assess oxidative stress in Raw264.7 cells according to the manufacturers' instructions.

### 2.10. Flow Cytometry

MitoSox assay was used to measure the level of oxidative stress in macrophages. Cells were collected and washed three times. After being stained with MitoSox (Invitrogen, USA) for 10 min, cells were washed again and prepared for flow cytometry analysis. The results were analyzed using FlowJo software (Tree Star).

### 2.11. Statistical Analysis

Statistical analysis was performed using GraphPad Prism 6.01 software (San Diego, CA, USA). All data were presented as mean ± standard error (SEM). Normally distributed data were compared using Student's *t*-test. *p* value < 0.05 was considered statistically significant.

## 3. Results

### 3.1. SS-31 Protects the Liver from Ischemia-Reperfusion Injury

**Figure 1:**
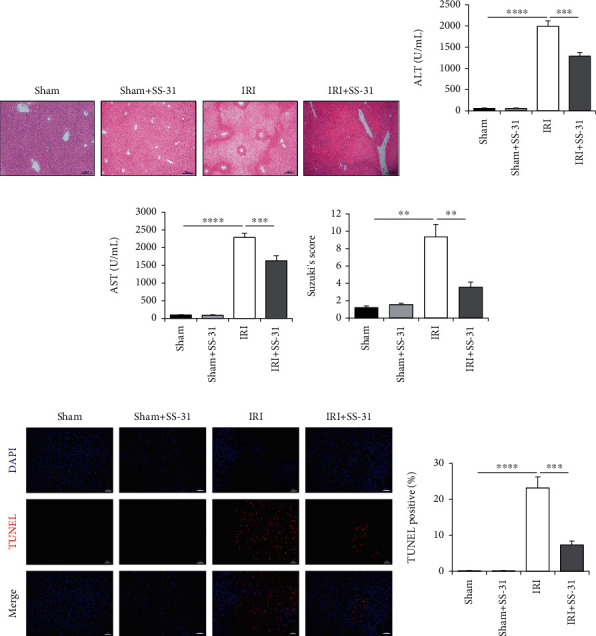
SS-31 treatment ameliorated liver ischemia-reperfusion injury. (a) Representative H&E staining of the liver section in different groups after IR (*n* = 4‐5 per group), scale bars, 200 *μ*m. (b, c) Serum ALT and AST of vehicle- and SS-31-treated mice were measured after IR procedure (*n* = 4‐5 per group). (d) Quantitative assessment of levels of liver injuries by using Suzuki's score. (e, f) Representative TUNEL staining of liver sections to assess the rate of apoptosis after IR with the administration of SS-31 (*n* = 4‐5 per group), scale bars, 100 *μ*m. Data are mean ± SEM, ^∗^*p* < 0.05, ^∗∗^*p* < 0.01, ^∗∗∗^*p* < 0.001, and ^∗∗∗∗^*p* < 0.0001 by unpaired Student's *t*-test.

To determine the protective effects of SS-31 against IRI, we first compared the liver damage at 6 h after hepatic IRI between vehicle-treated and SS-31-treated groups. In the vehicle group, the liver sections exhibited large areas of necrosis; the hepatocytes lost normal cellular morphology, and there was obvious inflammatory cell infiltration (Figure 1(a)). On the contrary, in the SS-31 group, liver injury was significantly alleviated. As shown in Figure 1(d), Suzuki's score was significantly lower in the SS-31 group compared with the vehicle group. Meanwhile, the levels of ALT and AST in serum were increased in the vehicle group and were significantly reduced in the SS-31 group (Figures 1(b) and 1(c)). Moreover, less TUNEL-positive cells were observed in the SS-31 group than the vehicle group (Figures 1(e) and 1(f)). These results suggested that SS-31 could protect liver damage from hepatic IRI.

### 3.2. The Oxidative Stress in Liver Is Decreased by SS-31 Treatment

**Figure 2:**
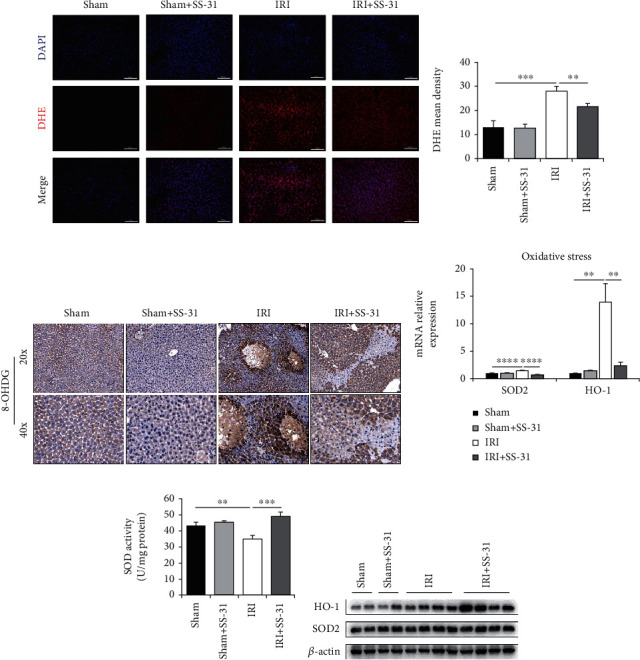
SS-31 treatment decreased oxidative stress in the liver after IRI. (a, b) Representative images of DHE staining in liver sections undergoing IRI treated with or without SS-31 (*n* = 4‐5 per group). (c) Representative 8-OHDG immunohistochemistry of liver sections with or without IRI (*n* = 4‐5 per group). (d) The mRNA levels of genes related to liver oxidative stress were measured. (e) The activity of MnSOD was detected by using the Superoxide Dismutase (SOD) Activity Assay Kit in the liver to assess liver oxidative stress (*n* = 4‐5 per group). (f) Immunoblot analysis of HO-1 and SOD2 expression in the liver (*n* = 4 per group). Scale bars, 100 *μ*m. Data are mean ± SEM, ^∗^*p* < 0.05, ^∗∗^*p* < 0.01, ^∗∗∗^*p* < 0.001, and ^∗∗∗∗^*p* < 0.0001 by unpaired Student's *t*-test.

Oxidative stress is an initial and critical factor during liver ischemia and reperfusion period. Since SS-31 has demonstrated antioxidant activity in various ischemia-reperfusion models, we also examined the level of oxidative stress after hepatic IRI in vehicle- or SS-31-treated groups. Consistently, both histology analysis and DHE staining showed significantly reduced liver oxidative stress level in the SS-31 group compared with the vehicle-treated group (Figures 2(a) and 2(b)). Also, the DNA oxidative damage marker, 8-OHDG, was significantly lower in the IRI+SS-31 group than that in the vehicle-treated group (Figure 2(c)). To further confirm the decreased oxidative stress in the SS-31 group, we also measured the expression levels of oxidative stress-related genes by qPCR. The results showed that increased HO-1 and SOD2 caused by IRI were significantly inhibited by SS-31 (Figure 2(d)); meanwhile, the other oxidative stress-related genes did not show obvious changes (Supplementary Figure 1). It is well known that the superoxide dismutases (SODs) are the major antioxidant enzymes, and SOD2 is also known as mitochondrial MnSOD [35]. Thus, we measured the activity of SOD2 in the liver and found that IRI significantly inhibited the activity of SOD2, and the inhibition was rescued by SS-31 administration (Figure 2(e)). We also noticed that the protein level of SOD2 in the liver did not change (Figure 2(f)). These results were consistent with the fact that SS-31 is an antioxidant peptide that targets mitochondria. The above data demonstrated that SS-31 treatment could significantly decrease the ROS production in the liver during IRI.

### 3.3. The Inflammation Response Is Mitigated by SS-31

**Figure 3:**
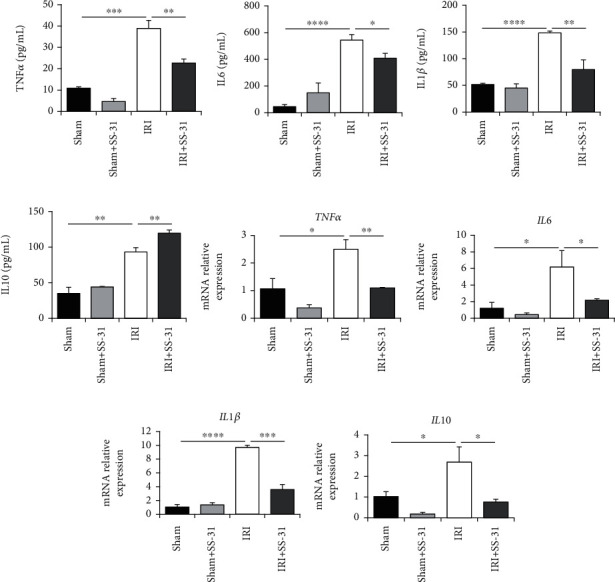
The production of inflammation-related cytokines is inhibited by SS-31 administration. (a–d) The levels of TNF*α*, IL6, IL1*β*, and IL10 in the serum of mice undergoing liver IRI were detected by the ELISA kit (*n* = 4 per group). (e–h) qPCR analysis of mRNAs encoding inflammation-related cytokines, including TNF*α*, IL6, IL1*β*, and IL10 in livers after IRI treated with or without SS-31 (*n* = 4 per group). Data are mean ± SEM, ^∗^*p* < 0.05, ^∗∗^*p* < 0.01, ^∗∗∗^*p* < 0.001, and ^∗∗∗∗^*p* < 0.0001 by unpaired Student's *t*-test.

In addition to oxidative stress, previous studies also revealed that inflammation response can contribute to liver damage after hepatic IRI. Therefore, we next tested the levels of inflammation cytokines in the serum, and the expression levels of cytokines in the liver. As we expected, the levels of proinflammation cytokines TNF*α*, IL1*β*, and IL6 decreased significantly in the serum of SS-31-treated group (Figures 3(a)–3(c)). We also measured the level of IL10, an essential anti-inflammation cytokine, and found that SS-31 treatment promoted the secretion of IL10 (Figure 3(d)). To further confirm the anti-inflammatory effects of SS-31, we measured the mRNA level of the above cytokines in the liver. As expected, the mRNA level of TNF*α*, IL6, and IL1*β* was consistent with their serum levels (Figures 3(e)–3(g)). Only the IL10 mRNA level was slightly different from its serum level (Figure 3(h)), and we will discuss it in the later session. These results suggested that the inflammation response was inhibited by SS-31 treatment.

### 3.4. SS-31 Inhibits the M1 Polarization of Kupffer Cells in the Liver

**Figure 4:**
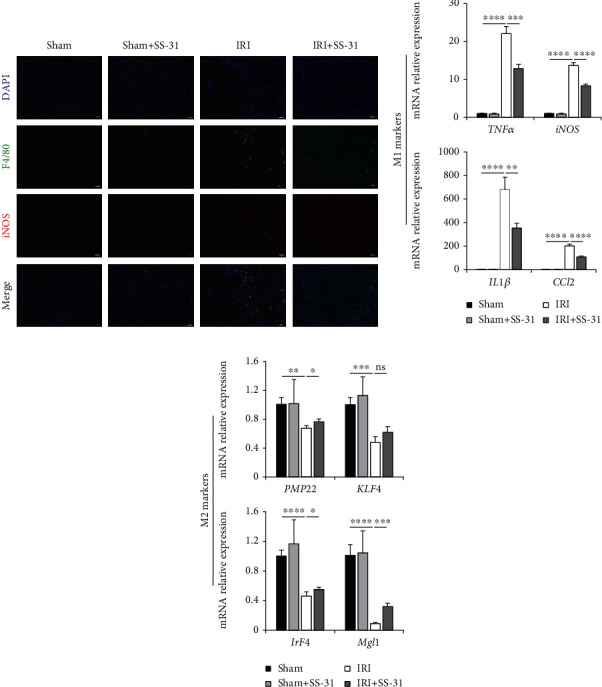
SS-31 inhibited the M1 polarization of KCs in the liver. (a) Representative immunofluorescence of iNOS (red) and F4/80 (green) in liver sections after IRI treated with or without SS-31 (*n* = 4‐5 per group). (b) Levels of mRNAs encoding TNF*α*, iNOS, IL1*β*, and CCL2 (M1 markers) in KCs (*n* = 4 per group). (c) Levels of mRNAs encoding PMP22, KLF4, IrF4, and Mgl1 (M2 markers) in KCs (*n* = 4 per group). Scale bars, 20 *μ*m. Data are mean ± SEM, ^∗^*p* < 0.05, ^∗∗^*p* < 0.01, ^∗∗∗^*p* < 0.001, and ^∗∗∗∗^*p* < 0.0001 by unpaired Student's *t*-test.

As demonstrated above, oxidative stress and inflammatory response were both enhanced during liver IRI. It is also believed that the excessive generation of ROS contributed to the cellular phenotype regulation of inflammatory macrophages, especially the M1 phenotype, which can promote the inflammation response during liver reperfusion. Hence, we hypothesized that SS-31 could suppress inflammation via modulating macrophage phenotypic transformation. To test this hypothesis, we performed immunofluorescence staining on liver tissues to examine the polarization of macrophages. Anti-F4/80 antibody was used to indicate macrophage/monocyte cells (green), anti-iNOS antibody was used to indicate M1 macrophages (red), and DAPI (blue) was used for nuclear staining. As shown in Figure 4(a), the SS-31-treated group and the sham group exhibited minimal macrophage activation and recruitment. In contrast, the vehicle-treated group showed obvious activation of macrophages and M1 polarization during IRI, and SS-31 treatment decreased the ratio of macrophage activation and M1 polarization. To further confirm this result, we isolated the KCs from the liver and measured the expression level of representative markers of M1-type and M2-type macrophages. The results corroborated our findings that SS-31 treatment decreased M1 macrophages and increased M2 macrophages during IRI (Figures 4(b) and 4(c)). Overall, these data suggested that SS-31 could inhibit the damage caused by inflammation response by modulating macrophage phenotype polarization.

### 3.5. SS-31 Downregulates Macrophage M1 Polarization *In Vitro*

**Figure 5:**
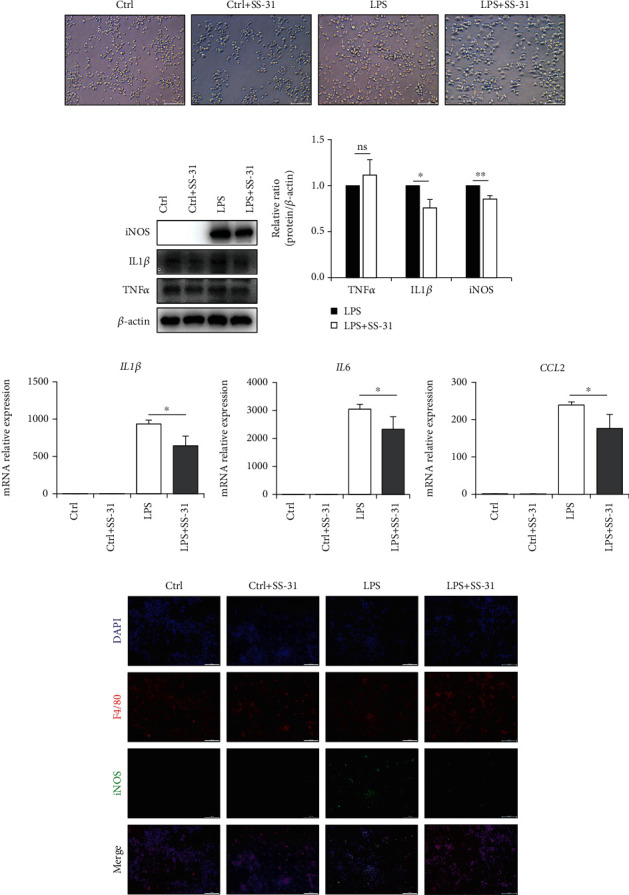
SS-31 downregulated LPS-induced macrophage polarization *in vitro*. (a) Representative images of the morphology of Raw264.7 cells stimulated with LPS and SS-31. Scale bars, 100 *μ*m. (b, c) Immunoblot analysis of inflammation-related proteins TNF*α*, IL1*β*, and iNOS in Raw264.7 cells treated with LPS and SS-31. (d–f) Levels of mRNAs encoding IL1*β*, IL6, and CCL2 (M1 markers) in Raw264.7 cells treated with LPS and SS-31. (g) Representative images of immunofluorescence of iNOS (green) and F4/80 (red) in Raw264.7 cells. Scale bars, 100 *μ*m. Data are mean ± SEM, ^∗^*p* < 0.05, ^∗∗^*p* < 0.01, ^∗∗∗^*p* < 0.001, and ^∗∗∗∗^*p* < 0.0001 by unpaired Student's *t*-test.

Next, we further explored the role of SS-31 in regulating macrophage M1 polarization *in vitro* using the Raw264.7 cell line. The change of macrophage morphology is one of the hallmarks of M1 polarization. Thereby, we first observed the morphology change of macrophage upon the stimulation of TLR4 ligand LPS. As shown in Figure 5(a), LPS greatly increased the ratio of polymorphic macrophages with a pseudopod, while SS-31 treatment led to more rounded cells, which were closer to shapes of M0 macrophage. iNOS staining is another visualized method to estimate the M1 macrophage polarization. Our result showed that the M1 phenotypic transformation was suppressed by SS-31 (Figure 5(g)). As mentioned above, M1 macrophage has proinflammatory functions; thus, measuring the expression of inflammation-related genes is an orthogonal way to assess the effect of SS-31 on macrophage polarization. We examined the classical inflammatory genes, TNF*α*, IL1*β*, and iNOS in Raw264.7 cells with different treatments (Figures 5(b) and 5(c)). Consistent with the above results, the LPS-induced expression of iNOS and IL1*β* was inhibited by SS-31 treatment. The gene expressions which indicate macrophage M1 polarization were almost in line with the previous findings, although the expression of TNF*α* and iNOS decreased upon SS-31 treatment, the differences were not statistically different (Supplementary Figure 2); on the other hand, the other markers including IL6, IL1*β*, and CCL2 were significantly downregulated (Figure 5(d), 5(e) and 5(f)). In summary, LPS-activated macrophage M1 polarization can be reversed by SS-31 treatment.

### 3.6. SS-31 Rescues LPS-Induced Mitochondrial Dysfunction and Oxidative Stress

**Figure 6:**
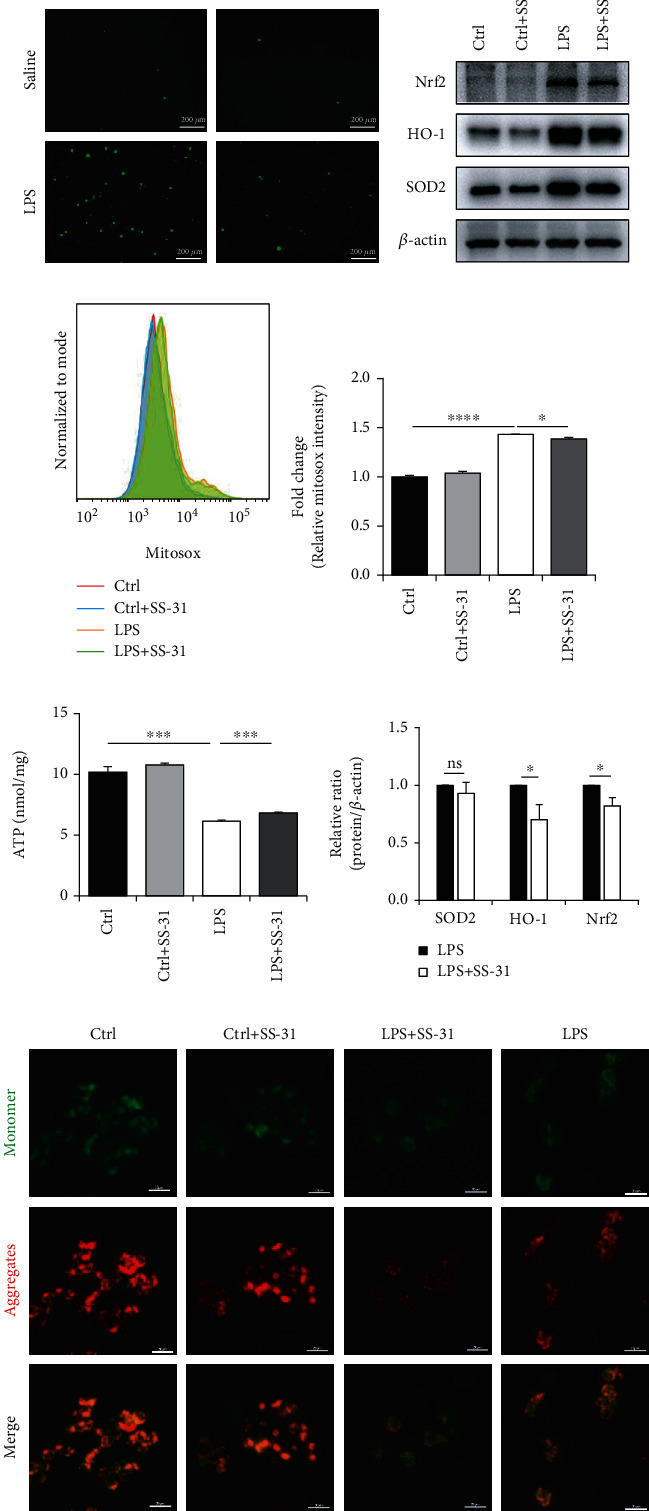
SS-31 preserved mitochondrial function and decreased the level of mtROS. (a) The changes of ROS levels of Raw264.7 cells were assessed by DCFH-DA staining, scale bars, 200 *μ*m. (b, f) Immunoblot analysis of Nrf2, HO-1, and SOD2 in Raw264.7 cells to evaluate the oxidative stress-related signaling changes. (c, d) Flow cytometry analysis of mtROS levels in Raw264.7 cells treating with LPS and SS-31 by staining with MitoSox. (e) The ATP levels were detected by the ATP Assay Kit to evaluate the mitochondrial function. (f) The staining of JC-1 in Raw264.7 cells was conducted to show the changes of mitochondrial membrane potential, scale bars, 20 *μ*m. Data are mean ± SEM, ^∗^*p* < 0.05, ^∗∗^*p* < 0.01, ^∗∗∗^*p* < 0.001, and ^∗∗∗∗^*p* < 0.0001 by unpaired Student's *t*-test.

To further investigate the SS-31-mediated regulation of M1 phenotypic transformation, we measured the level of oxidative stress in LPS-stimulated macrophages by examining DCFH-DA staining and the expression of antioxidant genes. DCFH-DA staining showed that the LPS-induced oxidative stress was reduced by SS-31 (Figure 6(a)). Consistently, the protein expression levels of HO-1, Nrf2, and SOD2 were also inhibited by SS-31 treatment (Figures 6(b) and 6(f)). As a mitochondrial-targeted peptide, the antioxidant activity of SS-31 is mainly through regulating the function and structure of mitochondria, which then leads to reduced mtROS. Therefore, we next determined the mtROS level of Raw264.7 cells by MitoSox staining. The results showed that SS-31 could slightly decrease LPS-induced mtROS (Figures 6(c) and 6(d)). To further validate the protective effects of SS-31 on mitochondria, we examined the ATP level of Raw264.7, which reflected the basic function of mitochondria. We found that SS-31 repaired the mitochondrial dysfunction caused by LPS and increased the production of ATP (Figure 6(e)). The mitochondrial membrane potential is another indicator for mitochondrial function, and the JC-1 staining showed that SS-31 could restore the mitochondrial membrane potential. Altogether, these results fully demonstrated that SS-31 could reduce the production of mtROS by maintaining the mitochondrial function, thereby suppressing oxidative stress induced by LPS.

### 3.7. SS-31 Regulates Macrophage Polarization through STAT1/3 Signals

**Figure 7:**
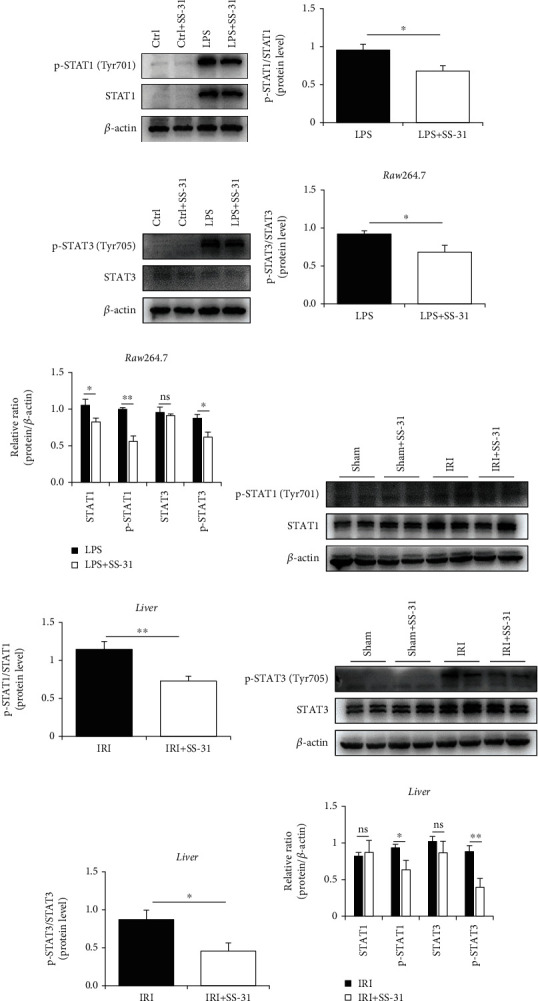
SS-31 regulated STAT1 and STAT3 to govern macrophage polarization. (a, b) Immunoblot analysis of STAT1 and p-STAT1 (Tyr701) in Raw264.7 treated with LPS with or without SS-31. (c, d) Immunoblot analysis of STAT3 and p-STAT3 (Tyr705) in Raw264.7 treated with LPS with or without SS-31. (e, f) Immunoblot analysis of STAT1 and p-STAT1 (Tyr701) in the liver after IRI with SS-31 pretreatment (*n* = 4 per group). (g, h) Immunoblot analysis of STAT3 and p-STAT3 (Tyr705) in the liver after IRI with SS-31 pretreatment (*n* = 4 per group). Data are mean ± SEM, ^∗^*p* < 0.05, ^∗∗^*p* < 0.01, ^∗∗∗^*p* < 0.001, and ^∗∗∗∗^*p* < 0.0001 by unpaired Student's *t*-test.

It has been reported that macrophage polarization is regulated in a STAT-dependent manner. Moreover, some studies showed that oxidative stress could modulate STAT phosphorylation [36]. Therefore, to better understand the mechanism underlying the regulation of SS-31 on macrophage polarization, we measured the protein levels of STAT proteins. Activation of STAT1 and STAT3 is known to play a critical role in regulating the release of proinflammation cytokines during acute injury [37]. Our results showed that SS-31 not only suppressed STAT1 and STAT3 expression but also inhibited the phosphorylation of STAT1 and STAT3 *in vitro* (Figures 7(a)–7(e)). We also found similar results in liver tissues (Figures 7(f)–7(j)). Overall, these findings indicated that SS-31 could regulate macrophage polarization in a STAT1/3-dependent manner.

## 4. Discussion

**Figure 8:**
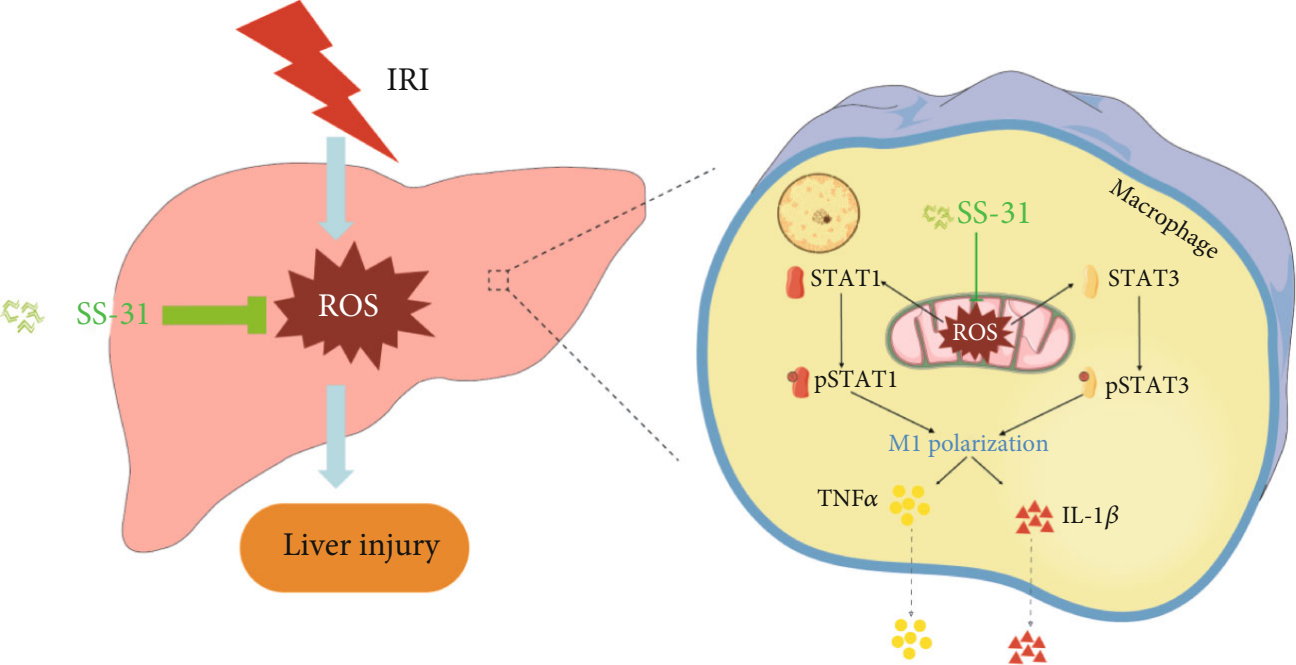
The diagram of the mechanism that SS-31 protects the liver against IRI. As a mitochondrial-targeted peptide, SS-31 preserves the function of mitochondrial and decreases the production of mtROS. With the reduction of mtROS, the phosphorylation process of STAT1 and STAT3 is inhibited, which in turn affects macrophage polarization to proinflammatory phenotype. Macrophages play a vital role in the inflammatory response caused by ischemia reperfusion, and inhibiting M1 polarization will reduce the release of inflammatory factors such as TNF*α* and IL1*β*. In general, SS-31 protects the liver from ischemia-reperfusion injury by regulating oxidative stress and inflammatory damage.

Ischemia-reperfusion injury is the most common complication in liver surgeries [38]. Over the last decades, many researchers have been looking for effective approaches to reduce ischemia-reperfusion injury. Currently, several methods have been demonstrated to protect the liver from hepatic IRI [39–41]. However, none of them has explored the role of antioxidant SS-31 in reducing hepatic IRI. Our work is the first to demonstrate the beneficial effects of SS-31 on alleviating hepatic IRI. According to our study, SS-31 treatment before surgery can stabilize mitochondrial function in macrophages, inhibit ROS production and STAT1/3 phosphorylation, and suppress the M1 polarization of macrophages. These effects can further lead to decreased inflammatory response, less ROS and proinflammation cytokine production, and ultimately protect the liver from IRI (Figure 8).

It has been known that oxidative stress is commonly involved in IRI. The excess production of ROS, which is largely generated from mitochondria, causes oxidative stress [42]. Excessive ROS leads to tissue damage and cell death by causing DNA damage, lipid peroxidation, and changes of protein structure or function [43]. Sometimes, it can serve as secondary messengers to regulate immune systems and maintain redox homeostasis [44]. The ROS-induced tissue damages are closely related to a variety of complications [45, 46].

Because of such adverse outcomes, many researches are trying to develop methods to reduce the excessive generation of ROS during IRI, and antioxidant is one of the effective ways. A variety of antioxidants have been experimentally tested in treating IRI. Moreover, it has been recently reported that melatonin [47], curcumin [48], stem cell [49], and nanoparticles [50] are all effective methods for treating or preventing IRI in multiple organs. As more and more studies are focused on SS-31 in IRI, it has become a potential treatment for IRI [26, 27]. In the present study, by examining the activity of SOD2 and the expression of 8-OHDG, we demonstrated that SS-31 was able to reduce oxidative stress during hepatic IRI. Moreover, this study highlighted the immunomodulation effect of SS-31 and its regulatory role in macrophage polarization, which may help us understand the mechanisms of its protective effect against IRI. Although our study verified the antioxidant properties of SS-31, we did not examine the changes of mitochondrial structure after SS-31 treatment; therefore, more evidence is needed to clarify the regulatory effects of SS-31 on mitochondrial function and the link between mitochondrial structure and function. Moreover, since SS-31 is an effective antioxidant peptide that has been verified in IRI of multiple organs, exploring new strategies to improve its therapeutic effect is also a major direction of future studies and the latest progress in nanomaterials may help with this development [50].

One of the most important discoveries of this study is the regulatory effects of SS-31 on macrophage phenotype. Macrophages are highly plastic immune cells involved in inflammation response during IRI [51]. There are generally two macrophage phenotypes, the classically activated macrophages (M1 polarization) and the alternatively activated macrophages (M2 polarization), which are classified based on their distinct roles in different microenvironments [52, 53]. Although numerous studies have investigated the mechanism of macrophage plasticity, the specific mechanisms are not fully understood because of their complex nature, especially the role of oxidative stress, which is the focus of this study. Several reports proposed that ROS was essential for the induction and maintenance of M1 macrophage polarization, and ROS can activate the MAPK signaling pathways [54] and NLRP3 inflammasome [55], which then upregulate the expression of proinflammation genes in macrophages. However, some other studies emphasized the impact of ROS production on M2 macrophage priming [56, 57]. In this study, we tested the expression of TNF*α*, iNOS, and IL1*β* in macrophages after SS-31 treatment and found that SS-31 could reduce the production of proinflammation cytokines and inhibit macrophage M1 polarization. This result is consistent with the findings from Yuan et al. that the inhibition of mitochondrial ROS prevented M1 polarization of macrophages by improving autophagic flux [58]. Further investigation is needed to clarify the role of ROS in macrophage polarization. In addition to the macrophages, the neutrophils and the hepatocytes are also vital contributors to hepatic IRI [59]; our data showed that the effect of SS-31 on the hepatocytes (Supplementary Figure 4a) and the neutrophils (Supplementary Figure 3a) may be also responsible for the protective effect of SS-31 on hepatic IRI. More efforts are required to clarify the beneficial effect of SS-31 on the liver.

In summary, we found that SS-31 could attenuate hepatic IRI by reducing macrophage M1 polarization and suppressing ROS and STAT1/3 signaling. Our findings suggest that SS-31 is a promising strategy for hepatic IRI prevention and also establish a foundation for future investigation on the protective effects of SS-31 against other liver disease caused by ROS. Although the preclinical data in the animal model support the efficacy of the SS-31, additional studies are needed to validate and expand our findings.

## Data Availability

The data used to support the findings of this study are available from the corresponding authors upon request.

## Supplementary Materials

Supplementary Materials Supplementary Table 1: the sequence of the primers in the article. Supplementary Figure 1: the mRNA levels of the other oxidative stress-related genes. Apart from the expressions of SOD2 and HO-1 were detected in the liver undergoing IRI (*n* = 4‐5 per group), we also detected the mRNA encoding SOD1 (a) and Gpx3 (b) to investigate the underlying mechanism of antioxidant capacity of SS-31; there was no significant difference between IRI and IRI+SS-31 groups in the expression of SOD1 and Gpx3. Supplementary Figure 2: the mRNA levels of TNF*α* and iNOS in Raw264.7 cells after treating with LPS and SS-31. To assess the inhibitory effect of SS-31 on M1 polarization, we detected the expression of M1 markers and found that TNF*α* (a) and iNOS (b) showed a slight drop after treating with SS-31, but the decrease did not reach statistical significance. Supplementary Figure 3: the infiltration of neutrophil in liver tissues after treating with SS-31. To assess the infiltration of other myeloid cells, immunohistochemical analysis of MPO-positive cells was conducted; the results showed that SS-31 treatment also inhibited the infiltration of neutrophil in liver tissues after IRI. Supplementary Figure 4: the protective effect of SS-31 on hepatocytes *in vitro*. To assess the role of SS-31 on hepatocytes, we established a cell model in vitro. AML12 cells were exposed to hypoxia condition (1% O_2_, 5% CO_2_, and 94% N_2_) at 37°C for 12 h; then, the medium was changed to the normal and the cells were cultured in normal condition (5% CO_2_, 95% air) at 37°C for 4 h. Western blot analysis of the expression of apoptosis protein (Bax, Bcl2, caspase3, cleaved caspase3, and PARP1) and oxidative stress protein (HO-1). The results showed a mild protection of SS-31 in hepatocytes.
