# Supplementary material for: SS-31 Protects Liver from Ischemia-Reperfusion Injury via Modulating Macrophage Polarization
Source: Oxid Med Cell Longev. 2021 Apr 13;2021:6662156. doi: 10.1155/2021/6662156 (PMC8057883; doi:10.1155/2021/6662156)
Supplement: Supplementary Materials — Supplementary Table 1: the sequence of the primers in the article. Supplementary Figure 1: the mRNA levels of the other oxidative stress-related genes. Apart from the expressions of SOD2 and HO-1 were detected in the liver undergoing IRI (n = 4‐5 per group), we also detected the mRNA encoding SOD1 (a) and Gpx3 (b) to investigate the underlying mechanism of antioxidant capacity of SS-31; there was no significant difference between IRI and IRI+SS-31 groups in the expression of SOD1 and Gpx3. Supplementary Figure 2: the mRNA levels of TNFα and iNOS in Raw264.7 cells after treating with LPS and SS-31. To assess the inhibitory effect of SS-31 on M1 polarization, we detected the expression of M1 markers and found that TNFα (a) and iNOS (b) showed a slight drop after treating with SS-31, but the decrease did not reach statistical significance. Supplementary Figure 3: the infiltration of neutrophil in liver tissues after treating with SS-31. To assess the infiltration of other myeloid cells, immunohistochemical analysis of MPO-positive cells was conducted; the results showed that SS-31 treatment also inhibited the infiltration of neutrophil in liver tissues after IRI. Supplementary Figure 4: the protective effect of SS-31 on hepatocytes in vitro. To assess the role of SS-31 on hepatocytes, we established a cell model in vitro. AML12 cells were exposed to hypoxia condition (1% O2, 5% CO2, and 94% N2) at 37°C for 12 h; then, the medium was changed to the normal and the cells were cultured in normal condition (5% CO2, 95% air) at 37°C for 4 h. Western blot analysis of the expression of apoptosis protein (Bax, Bcl2, caspase3, cleaved caspase3, and PARP1) and oxidative stress protein (HO-1). The results showed a mild protection of SS-31 in hepatocytes. [file 6662156.f1.docx]

Supplementary Information

**Supplementary Table1: The sequence of the primers in the article**

| GENE | SPECIES | Forward Primer | Reverse Primer |
| --- | --- | --- | --- |
| ACTB | Mus | GGCTGTATTCCCCTCCATCG | CCAGTTGGTAACAATGCCATGT |
| TNFa | Mus | GGTGCCTATGTCTCAGCCTCTT | GCCATAGAACTGATGAGAGGGAG |
| iNOS | Mus | GTTCTCAGCCCAACAATACAAGA | GTGGACGGGTCGATGTCAC |
| IL6 | Mus | TACCACTTCACAAGTCGGAGGC | CTGCAAGTGCATCATCGTTGTTC |
| IL1b | Mus | TGGACCTTCCAGGATGAGGACA | GTTCATCTCGGAGCCTGTAGTG |
| CCl2 | Mus | TAAAAACCTGGATCGGAACCAAA | GCATTAGCTTCAGATTTACGGGT |
| PMP22 | Mus | CATCGCGGTGCTAGTGTTG | GATCAGTCGTGTGTCCATTGC |
| IL10 | Mus | CTTACTGACTGGCATGAGGATCA | GCAGCTCTAGGAGCATGTGG |
| Mgl1 | Mus | CAATGTGGTTAGTTGGATCGGC | CCCAGTTCTTAAAGCCTTTCTCA |
| Klf4 | Mus | GGCGAGTCTGACATGGCTG | GCTGGACGCAGTGTCTTCTC |
| Irf4 | Mus | CCGACAGTGGTTGATCGACC | CCTCACGATTGTAGTCCTGCTT |
| HO-1 | Mus | AGGTACACATCCAAGCCGAGA | CATCACCAGCTTAAAGCCTTCT |
| SOD2 | Mus | CAGACCTGCCTTACGACTATGG | CTCGGTGGCGTTGAGATTGTT |


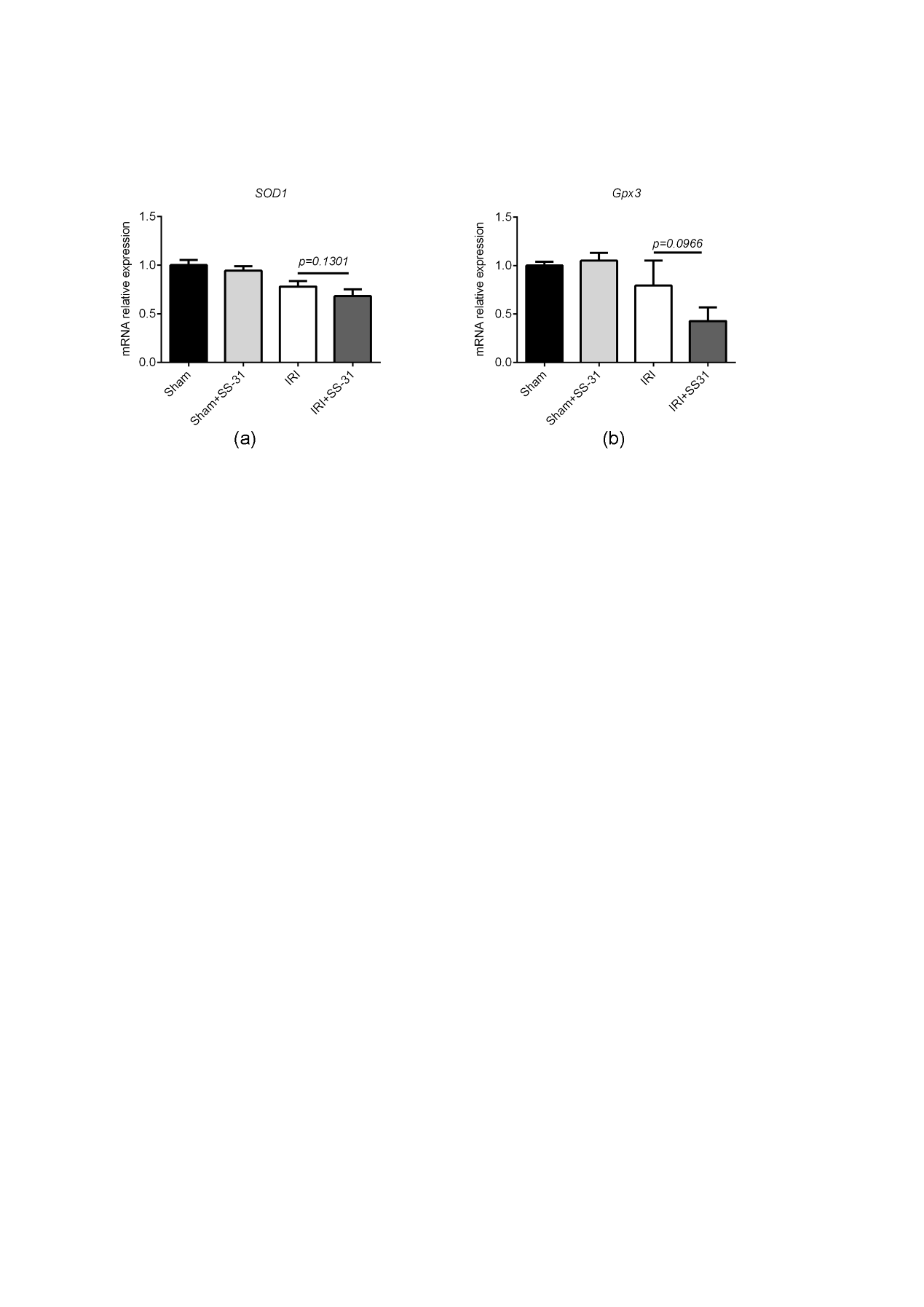


**Supplementary Figure1: The mRNA levels of the other oxidative stress-related genes.** Apart from the expressions of SOD2 and HO-1 were detected in liver undergoing IRI (n=4-5 per group), we also detected the mRNA encoding SOD1 (a) and Gpx3 (b) to investigate the underlying mechanism of antioxidant capacity of SS-31, there were no significant difference between IRI and IRI+SS-31 group in the expression of SOD1 and Gpx3.


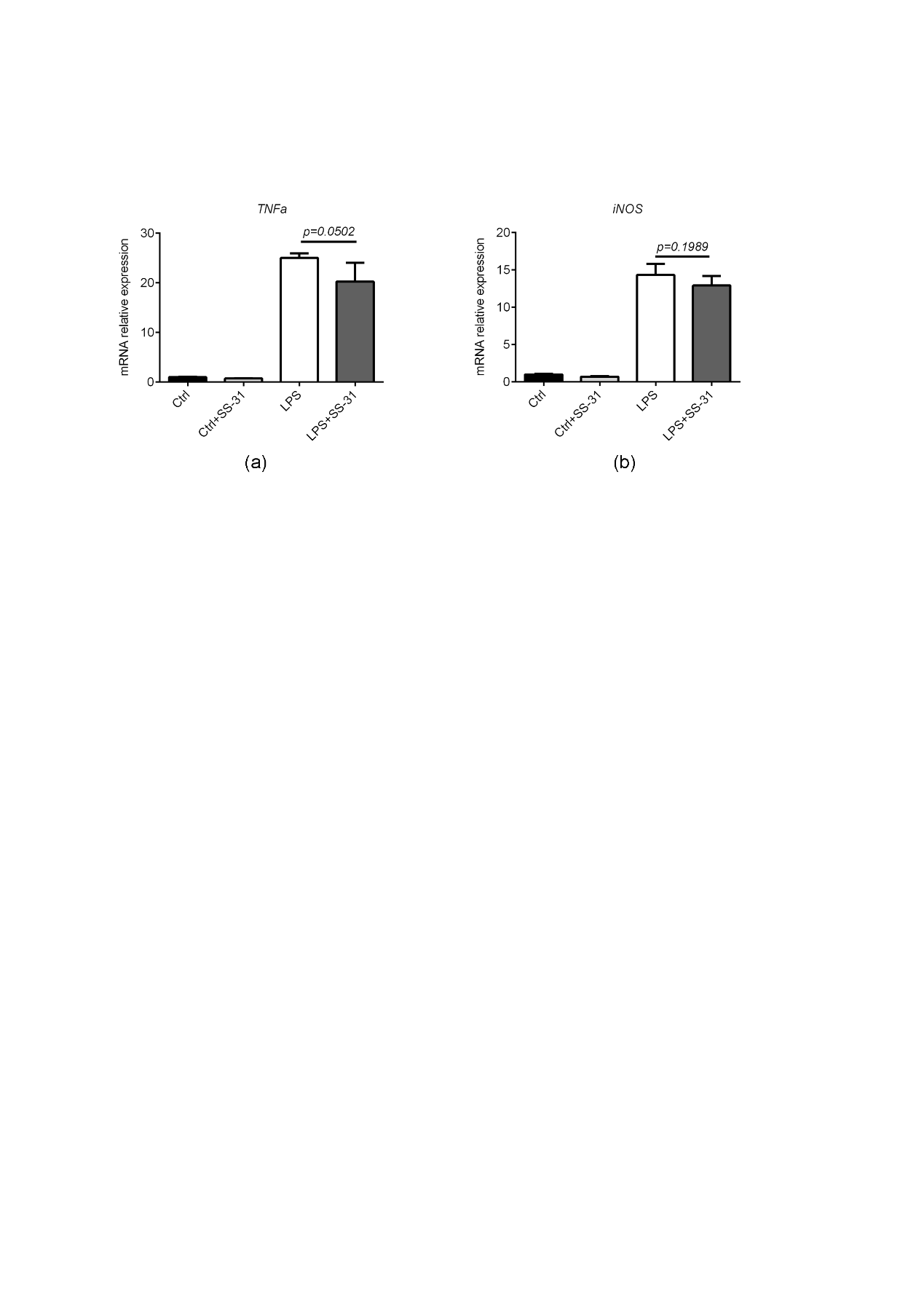


**Supplementary Figure2: The mRNA levels of TNFa and iNOS in RAW264.7 cells after treating with LPS and SS-31.** To assess the inhibitory effect of SS-31 on M1 polarization, we detected the expression of M1 markers and found that TNFa (a) and iNOS(b) showed a slight drop after treating with SS-31, but the decrease didn’t reach statistical significance.

**
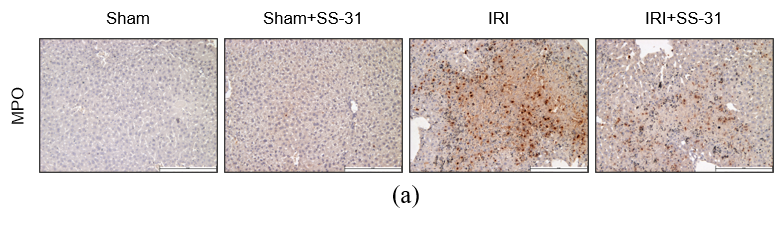
**

**Supplementary Figure3: The infiltration of neutrophil in liver tissues after treating with SS-31.** To assess the infiltration of other myeloid cells, immunohistochemical analysis of MPO-positive cells were conducted, the results showed that SS-31 treatment also inhibited the infiltration of neutrophil in liver tissues after IRI.

**
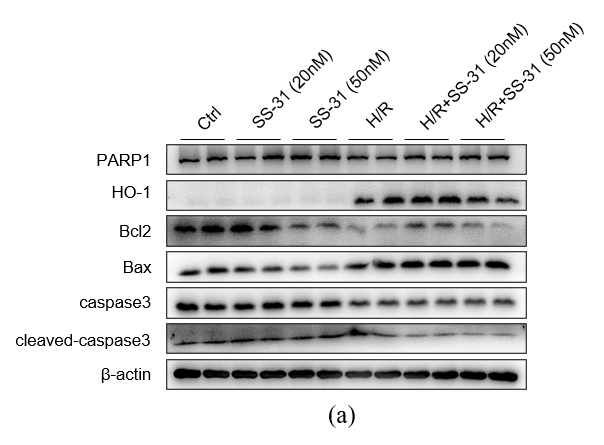
**

**Supplementary Figure4: The protective effect of SS-31 on hepatocytes *in vitro*.** To assess the role of SS-31 on hepatocytes, we established a cell model in vitro. AML12 cells were exposed to hypoxia condition (1%O_2_, 5%CO_2_, 94%N_2_) at 37℃ for 12h; then, the medium was changed to the normal and the cells were cultured in normal condition (5%CO_2_, 95%air) at 37℃ for 4 h. Western blot analysis of the expression of apoptosis protein (Bax, Bcl2, caspase3, cleaved caspase3, PARP1) and oxidative stress protein (HO-1). The results showed a mild protection of SS-31 in hepatocytes.
